# Social distance and anonymity modulate fairness consideration: An ERP study

**DOI:** 10.1038/srep13452

**Published:** 2015-08-21

**Authors:** Rongjun Yu, Pan Hu, Ping Zhang

**Affiliations:** 1 School of Psychology and Center for Studies of Psychological Application, South China Normal University, Guangzhou, China; 2 Department of Psychology, National University of Singapore, Singapore; 3 Singapore Institute for Neurotechnology (SINAPSE), Center for Life Sciences, National University of Singapore, Singapore; 4 Neurobiology/Ageing Programme, Center for Life Sciences, National University of Singapore, Singapore

## Abstract

Previous research indicated that fairness consideration can be influenced by social distance. However, it is not clear whether social distance and anonymity have an interactive impact on fairness evaluation during asset distribution and whether these processes can be documented in brain activity. Using a modified ultimatum game combined with measures of event related potential (ERP), we examined how social distance and anonymity modulate brain response to inequality. At the behavior level, we found that acceptance rate and reaction time can be substantially modified by social distance and anonymity. Feedback-related negativity, an ERP component associated with conflict between cognitive and emotion motives, was more negative in response to unfairness than fairness from strangers; however, it showed an opposite trend for unfair offers provided by friends, suggesting that the influence of social distance on fairness perception is relatively fast. The P300 in response to fair offers was more positive when the proposers made offers when uncertain about partner identity than when certain about partner identity. These results suggest that unfairness is evaluated in a fast conflict detection stage and a slower stage that integrates more complex social contextual factors such as anonymity.

Human cooperation requires specific mechanisms that balance individuals’ self-interest with concern and respect for others. Individuals try to ensure fairness to others in daily life, and hope to get fair treatment in return. By contrast, we may get angry and sometimes punish others when faced with unfairness, even when this results in a loss for us^1^. From an evolutionary perspective, this pattern of behavior can minimize the probability of similar future harm. Previous research showed that people’s fairness consideration was influenced not only by the balance between their own and others’ gains^2^,^3^ but also by contextual factors, such as informational asymmetry between proposer and recipient^4^,^5^, intentions of the interaction partner^6^, and social distance^7^,^8^.

Among these contextual factors, social distance has a significant impact on fairness consideration. In the relational models theory, Fiske created a taxonomy of relationships for making sense of others’ social behavior^9^. According to this theory, people use four cognitive models to coordinate social relationships and generate social expectations. In the communal sharing relationships, people treat material objects as things they have in common and do not attend to how much each group member contributes or receives—simple membership in the group is sufficient to entitle an individual member to use resources the group controls. In market pricing relationships, people take a calculatingly rational approach to social life and make decisions based on calculations of cost-benefit ratios in self-interested exchange. Equality matching relationships emphasize the obligation to reciprocate and the equal value of persons, whereas authority ranking relationships allow high-ranking people to preempt valuable items in distributions. Thus, only in communal sharing, personal relationships among people play a key role in determining individuals’ behaviors in social exchange. The influence of social distance on economic behavior has recently become a focus of considerable research. For example, the script theory argues that individuals have scripts for both normal economic transactions and transactions among friends^10^,^11^. Previous research has demonstrated that social distance influences people’s justice consideration^12^. In particular, individuals hold different justice expectations toward friends and strangers and are more motivated to agree on offers with friends than with strangers. Peters and van den Bos extended this perspective by showing that when people’s interaction partners are friends, people are indeed more satisfied with being underpaid and less satisfied with being overpaid, compared to when their partners are unknown others. These studies indicate that justice is especially important to people when they are in an exchange relationship, as opposed to being in a communal relationship^13^. Other research showed that when deciding for a stranger, individuals were less likely to reject unfair offers, suggesting that social distance transfers an exchange relationship to a market pricing relationship and thus decreases the sensitivity to fairness^14^.

Research on economic games adds to the evidence that anonymity is another significant factor influencing human behavior, consistent with people’s other-regarding preference^15^,^16^. The standard procedure in experimental economics maintains anonymity among laboratory participants, while many field interactions are conducted with neither complete anonymity nor complete familiarity. Whether the identity of the other player is identifiable may influence social behaviors. Research using the dictator game has been shown that that when family names of their counterparts were revealed, dictators allocated a significantly larger portion of the pie to the counterpart, suggesting that even some degree of anonymity can modulate fairness concerns^16^. Two recent event related potential (ERP) studies investigated the neural responses to unfair offers from friends and strangers but found conflicting findings^7^,^17^. One possible explanation is that partner identity is clearly identifiable to participants in one study^17^, but it is not identifiable in the other study^7^. The above findings suggest a significant role of social distance and anonymity in fairness consideration. However, few studies have investigated under what circumstances these different factors work independently when they are manipulated concurrently.

In the current study we used a modified UG game combined with measures of ERP to investigate further to what extent related neurophysiological responses to justice concerns can be modulated by social distance and anonymity. We manipulated anonymity by changing the certainty about the other player’s identity. In this study, participants played an adapted version of the Ultimatum Game, believing that they were facing four proposers: two accompanying friends and two strangers. In the certainty about identity condition, participants were certain of the identity based on the proposer’s image and name shown to participants. In the uncertainty about identity condition, participants were informed that they were interacting with one of their friends or one of the strangers without knowing exactly which one it actually was. Thus, there were four experimental conditions: certainty-friend (certainty about friend’s identity); uncertainty-friend (uncertainty about friend’s identity); certainty-stranger (certainty about stranger’s identity); uncertainty-stranger (uncertainty about stranger’s identity). Although the players in the uncertainty about identity condition were not truly anonymous, they were indeed not fully identifiable to participants. Participants were told that all players in the game were aware of the manipulation of anonymity.

The purpose of our study was thus twofold: (1) to investigate how the brain responds differentially to fair and unfair offers in UG; and more importantly (2) to examine how the social distance between the proposer and recipient and the degree of anonymity modulate the recipient’s brain response to different offers. Previous studies have investigated brain responses to fair and unfair offers by using the ultimatum game^18^,^19^,^20^. These studies consistently found that when the proposals were presented to recipients, the feedback related negativity (FRN) amplitude was more pronounced for unfair offers compared to fair offers^18^,^19^,^20^, and the effect was most pronounced for subjects with high concern for fairness. FRN is a negative deflection at fronto-central recording sites that peaks between 250 and 300 ms post onset of outcome feedback^21^. Source localization analysis has shown that FRN is generated at the ACC^21^,^22^, a region that may reflect the conflict between cognition and emotion^23^. The FRN has been shown to be sensitive to the valence of outcomes. It is more pronounced for negative feedback associated with unfavorable outcomes, such as incorrect responses or monetary loss, than for positive feedback, and more pronounced for “worse than expected” prediction errors than prediction congruence^21^,^22^,^24^,^25^,^26^,^27^.

Another related ERP component is the P300; it is the most positive peak in the 200–600 ms time window post onset of feedback and has the most positive deflection at posterior electrode locations. Previous research employing the oddball paradigm suggested that the P300 is related to higher-order cognitive operations, such as selective attention and resource allocation^28^, specifically in response to unexpected (low probability) stimuli^29^,^30^,^31^. It is also suggested that the P300 is sensitive to a later, top-down controlled process of outcome evaluation, where factors related to the allocation of attentional resources come into play. Those factors include reward valence, reward magnitude, and magnitude expectancy^32^.

Taking the above studies together, we focused on the FRN and the P300 responses to offers in the ultimatum game (Fig. 1). We predicted that the FRN would be modulated by the social distance and the degree of anonymity. Individuals extend their justice concern more to their friends than to strangers, making justice more important in the context of friendship than in interactions with strangers, and unfair offers from friends should lead to stronger social norm violation than unfair offers by strangers. But under anonymous conditions, with no social identification, the unfair offer should lead to weaker social norm violation than an unfair offer made in a certainty about identity condition. We expected this to be detected in the recipient’s brain activity at an early stage of evaluative processing, possibly indexed by the amplitudes of FRN. For the P300, we predicted that it would be more positive for fair offers than unfair offers. Because in anonymous conditions more attention and resources would need to be distributed in confirming the proposer’s identity, we predicted that P300 would be more positive in uncertainty about identity conditions than in certainty about identity conditions. Based on the research to date, it is not yet clear how P300 would be modulated by the manipulation of social distance.

## Results

The post-experiment questionnaire indicated that all nineteen participants generally believed the setup of the experiment, with mean ± SE of 5.176 ± 0.260 (with 1 indicating “do not believe at all” and 7 indicating “truly believe”) for the questionnaire. A manipulation check of social distance, using participant self-reports on the IOS, showed that these participants had a generally close relationship with their friends. Participants’ self-reports on IOS confirmed that they had a closer relationship with their friends (mean ± SE, 5.000 ± 0.358) than with strangers (mean ± SE, 1.315 ± 0.109), t(18) = 10.500, p < 0.001. Similarly, on trust scale, participants showed stronger trust with their friends (mean ± SE, 83.578 ± 2.949) and with strangers (mean ± SE, 64.210 ± 2.280), t(18) = 4.876, p < 0.001. No significant correlation between the scale scores with the ERP amplitudes was found.

Acceptance rates for different division schemes are presented in Fig. 2A. A 2 (fairness level: fair vs. unfair offer) × 2 (social distance: friend vs. stranger) × 2 (degree of anonymity: certainty about identity vs. uncertainty about identity) repeated-measure ANOVA was performed to analyze the data. Acceptance rate was defined by the percentage of acceptance choices in each condition. Results revealed the main effect of fairness was significant, F(1, 18) = 25.016, p < 0.001, *η*_*p*_^2^ = 0.582, with the acceptance rate for fair offers (mean ± SE, 98.5% ± 0.4%) being higher than that for unfair offers (mean ± SE, 70.4% ± 5.6%). The main effect of social distance was also significant F(1, 18) = 8.503, p < 0.01, *η*_*p*_^2^ = 0.321; the acceptance rate for offers from friends (mean ± SE, 88.5% ± 2.8%) was higher than that for offers from strangers (mean ± SE, 80.4% ± 3.5%). The main effect of anonymity was significant, F(1, 18) = 10.446, p < 0.01, *η*_*p*_^2^ = 0.367, with the acceptance rate for the offers in the certainty about identity condition (mean ± SE, 85.3% ± 2.9%) being higher than that for the offers in the anonymous condition (mean ± SE, 83.7% ± 2.8%). The interaction between social distance and fairness level was significant, F(1, 18) = 10.059, p < 0.01, *η*_*p*_^2^ = 0.358. Unfair offers from friends were accepted more often (mean ± SE, 78.6% ± 5.4%) than unfair offers from strangers (mean ± SE, 62.1% ± 6.9%), t(18) = 3.057, p < 0.01. In the fair condition, there was no significant difference on acceptance rate for fair offers from friends (mean ± SE, 98.3% ± 0.5%) vs. from strangers (mean ± SE, 98.5% ± 0.3%), t(18) = −0.377, p > 0.2. In other words, within the stranger condition, unfair offers were rejected more often than fair offers (62.1% vs. 98.5%), whereas within the friends condition, this effect was less pronounced (78.6% vs. 98.3%). The results suggest that the recipient was particularly tolerant of unfairness coming from a friend in the unfair condition. No other effect was significant, p > 0.2.

A repeated-measure ANOVA was also performed to analyze reaction times (see Fig. 2B) with respect to social distance, anonymity and response (accepted fair offers vs. rejected unfair offers). The reaction times for accepted unfair offers and rejected fair offers were not analyzed as they were under-represented. We found a significant main effect of social distance F(1, 18) = 8.364, p < 0.05, *η*_*p*_^2^ = 0.317, with recipients making a faster response to strangers’ offers (mean ± SE, 776 ms ± 35) than friends’ offers (mean ± SE E, 859 ms ± 44). There was also a significant main effect of response, F(1, 18) = 14.201, p < 0.01, *η*_*p*_^2^ = 0.441, with overall faster acceptance of fair offers than rejection of unfair offers (mean ± SE, 747 ms ± 32 vs. 887 ms ± 49), while there was a significant interaction between social distance and response, F(1, 18) = 10.962, p < 0.01, *η*_*p*_^2^ = 0.379. Post-hoc analysis revealed that the RT difference between fair (mean ± SE, 747 ms ± 32) and unfair offers (mean ± SE, 887 ms ± 49) was larger in the friend condition (mean ± SE, 228 ms ± 60) than in the stranger condition (mean ± SE, 96 ms ± 15), t(18) = 2.397, p < 0.05. No other effect was significant, p > 0.2.

Post-experiment emotion ratings for 8 conditions are shown in Fig. 3. For the self-reported satisfaction measure, repeated-measures ANOVA using social distance (friend vs. stranger), fairness (fair vs. unfair), and anonymity (certainty about identity vs. uncertainty about identity) as independent factors found a significant main effect of fairness, F(1, 18) = 50.793, p < 0.001, *η*_*p*_^2^ = 0.738, with fair offers rated as more satisfactory (mean ± SE, 9.296 ± 0.274) than unfair offers (mean ± SE, 6.066 ± 0.475). The interaction between fairness and social distance was also significant, F(1, 18) = 5.171, p < 0.05, *η*_*p*_^2^ = 0.223. Post-hoc analysis revealed that the satisfaction difference between fair (mean ± SE, 9.296 ± 0.274) and unfair offers (mean ± SE, 6.066 ± 0.475) was larger in the stranger condition (mean ± SE, 3.802 ± 0.629) than in the friend condition (mean ± SE, 2.657 ± 0.376), t(18) = −2.274, p < 0.05, suggesting that social distance modulates self-reported satisfaction of outcome. No other effect was significant, p > 0.2.

For self-reported fairness, reported-measure ANOVA revealed that the main effect of fairness was significant, F(1, 18) = 70.122, p < 0.001, *η*_*p*_^2^ = 0.796: Fair offers were rated as more positive (mean ± SE, 9.566 ± 0.186) than unfair offers (mean ± SE, 5.921 ± 0.432). The main effect of social distance was also significant, F(1, 18) = 5.207, p < 0.05, *η*_*p*_^2^ = 0.224, showing that offers from friends were rated as more positive (mean ± SE, 8.039 ± 0.201) than offers from strangers (mean ± SE, 7.447 ± 0.347), suggesting that social distance modulates the recipient’s fairness consideration. No other effect was significant, p > 0.2.

For the self-reported surprise in response to outcomes, repeated-measures ANOVA revealed a significant interaction between anonymity and fairness level, F(1, 18) = 5.107, p < 0.05, *η*_*p*_^2^ = 0.221. Post-hoc analysis revealed that the surprise difference between fair and unfair offers was larger in the certainty about identity condition (mean ± SE, 0.934 ± 0.827) than in the uncertainty about identity condition (mean ± SE, −0.026 ± 0.787), t(18) = 2.260, p < 0.05. We also found a significant interaction between social distance and fairness level, F(1, 18) = 10.029, p < 0.01, *η*_*p*_^2^ = 0.358. Post-hoc analysis revealed that the surprise difference between fair (mean ± SE, 4.079 ± 0.822) and unfair (mean ± SE, 4.533 ± 0.533) offers was larger in the friend condition (mean ± SE, 2.394 ± 1.013) than in the stranger condition (mean ± SE, −1.486 ± 0.968), t(18) = 3.167, p < 0.01. These results suggest that the recipient was particularly surprised by an unfair offer coming from a friend.

Each of the 19 participants had at least 36 trials in each condition for EEG averaging. The group waveforms for the four experimental conditions after 1–20 HZ band-pass filtering are shown in Fig. 4A,B. At the frontal-central locations, for the FRN amplitude (see Fig. 5), repeated-measures ANOVA using social distance (friend vs. stranger), fairness (fair vs. unfair), and anonymity (certainty about identity vs. uncertainty about identity) as independent factors, found no main effects but did find a significant interaction between social distance and fairness, F(1, 18) = 4.642, p = 0.045, *η*_*p*_^2^ = 0.205. Post-hoc analysis revealed that the FRN effect (unfair minus fair) was larger in the friend condition (mean ± SE 0.210 μV ± 0.295) than in the stranger condition (mean ± SE, −0.509 μV ± 0.266), t(18) = 2.159, p = 0.045. There was also an opposite trend. Unfair offers elicited more negative FRN than fair offers in the stranger-allocation, while unfair offers elicited more positive FRN than fair offers in the friend-allocation. No other effect reached significance, p > 0.1. The difference waveforms and corresponding topographical maps are shown in Fig. 6.

The group waveforms for the four experimental conditions after 20 Hz low-pass filtering are shown in Fig. 4C,D. For the P300 amplitude (see Fig. 5), An ANOVA on the peak amplitudes over the central-posterior electrodes, with anonymity, social distance and fairness as three within-subject variables, revealed a significant main effect of anonymity level, F(1, 18) = 10.862, p < 0.01, *η*_*p*_^2^ = 0.376, with uncertainty about identity allocation (mean ± SE, 9.352 μV ± 1.278) eliciting more positive responses than certainty about identity allocation (mean ± SE, 7.425 μV ± 0.977). The interaction between anonymity and fairness level was significant, F(1, 18) = 5.060, p < 0.05, *η*_*p*_^2^ = 0.219. Post-hoc analysis revealed that the P300 effect (unfair minus fair) was larger in the anonymous condition (mean ± SE, 0.307 μV ± 0.504) than in the certainty about identity condition (mean ± SE, −0.833 μV ± 0.505), t(18) = 2.122, p = 0.048. No other effect was significant, p > 0.2.

## Discussion

Our study demonstrated that social distance and anonymity influence recipients’ behavioral reactions as well as their brain responses to unequal asset allocation schemes in the UG. We found that participants were more likely to reject not only unfair offers from strangers compared to friends but also unfair offers under the anonymous condition compared to the certainty about identity condition. At the neural level, unfair offers allocated by strangers elicited more negative going ERP responses compared to fair offers in an early time window (250–350 ms). However, the FRN showed an opposite trend for unfair offers provided by friends, suggesting that the influence of social distance on fairness perception is relatively fast. Our data provide separate information about the different effects due to social distance and identity by demonstrating the FRN is strongly responsive only to social distance. In a late time window (350–500 ms), the P300 in response to fair offers was more positive when the proposers made offers in the anonymous condition than in the certainty about identity condition. These results suggest that unfairness is evaluated in a fast conflict detection stage and a slower stage that integrates more complex social contextual factors such as anonymity.

Our behavior data not only conform to previous findings on inequity aversion and altruistic punishment when interacting with an unknown proposer^1^,^33^, but also demonstrate how social distance affects subjective perception of justice, reflected in the rejection rate and recipients’ fairness ratings. According to Halpern (1994, 1997), people use different scripts when conducting economic transaction with friends compared with strangers. Friendship provokes a fundamental bias regarding friends’ unfair offers; therefore, participants might have accepted unfair offers as a way to weaken potential disappointment and conflict after the end of the game. When confronted with unfair treatment from the stranger, the negative emotion induced by the feeling of being betrayed may trigger participants’ willingness to enact punishment, which in turn protects them from future similar harm.

Bohnet and Frey posited a hierarchy of “institutional characteristics” that determines the extent to which fairness considerations are active^34^. With anonymity, one has only purely intrinsic motivation to behave fairly; when people can identify each other, the fairness norm is partially activated^34^. By the same logic, in the anonymous condition participants instinctively prefer to punish the unfair proposer by sending a negative judgment of unfairness, which in turn would protect them from potential future damage. In addition, without experiencing the negative emotion associated with making the decision to reject a proposal for lack of sufficient social interaction information, they can behave inconsistently over the course of several trials. However, in the certainty about identity condition, the fairness norm is activated by the presented visual information with which participants can identify each other. They then have to take social feedback and social consequences into consideration when trying to conform to the social fairness norm, such as being in an awkward position or losing the friendship, which may result in the rejection difference between the two conditions.

Our ERP findings are in line with the study by Campanhã *et al.*^17^, in which the amplitude of the FRN was modulated by the social distance between the recipient and proposer; the FRN typically associated with unfair offers was reversed to positive polarity when the proposer was a friend rather than an unknown person. However, Wu *et al.* found a different result by manipulating the social distance in the dictator game, which indicated that FRN was more negative-going in response to unfair than to fair offers from friends whereas it did not show differential responses to offers from strangers^7^. We suspect that the discrepancy between the two studies may be the result of employing different paradigms (the ultimatum game vs. the dictator game) and different degrees of anonymity. Unlike recipients in the ultimatum game who have retaliatory power, participants in the dictator game are completely powerless and fully depend on the allocator for their outcome. Previous research has shown that recipients expect higher outcomes when they have more retaliatory power^35^. It is possible that participants in the dictator game expected that friends would give them fairer offers than strangers when they were in powerless situations. Such difference might be reduced in the ultimatum game, as the recipients may think that their friends would be less worried about unfair offers being rejected because of the close relationship between the two players. But in the dictator game, the recipients may think that strangers would give them more than friends since strangers would be worried about recipients’ retaliatory power. In the communal sharing relationships, justice concerns are less important for in-group members than for strangers. Thus, when playing with strangers, social expectations change dramatically from the dictator game (without punishment threat) to ultimatum game (with retaliatory power) but expectations change little across games when playing with friends.

Another important difference between the two studies is the degree of anonymity. Wu *et al.* manipulated anonymity such that participants were uncertain about the identity of their partners^7^, whereas Campanhã *et al.* used a complete certainty about identity condition^17^. Our study found that degree of anonymity did not modulate the FRN patterns in response to unfairness. Thus, the discrepancies between the two studies are more likely due to the different paradigms used. We observed that friends’ unfair offers elicited positive amplitudes, whereas unfair offer from strangers elicited negative amplitudes. Although this difference did not reach statistical significance, it was consistent with our prediction that social distance modulates the recipient’s fairness consideration.

The FRN is generally assumed to originate from the anterior cingulate cortex (ACC) or the medial frontal cortex^21^. The medial frontal cortex generates several ERP components associated with performance monitoring, such as the error-related negativity (ERN), the N200 (N2), and the FRN. Some researchers have also used the term medial frontal negativity (MFN) to describe specifically the FRN^7^,^17^,^21^. Botvinick *et al.* argued that ACC activity can be linked to the detection of cognitive conflict and may mirror the conflict between cognitive and emotional motivation in the ultimatum game^23^. Camerer *et al.* extended this finding by demonstrating that the ACC may struggle to resolve the conflict between wanting to accept money because of its planned reward value and disliking the ‘disgust’ of being treated unfairly^3^. In our task, participants would experience a stronger conflict because they received unfair personal gain at the expense of hurting themselves when confronted with the stranger’s unfair proposal. However, for the friend’s unfair offer, this kind of conflict would be suppressed to protect the friendship and monetary gain that could be divided after completing the experiments. The impact of the midbrain dopamine signals on ACC, which generates the FRN, can reflect the conflict between cognition and emotion, reflected in the amplitude of the FRN.

It has been claimed that the FRN represents a reward prediction error, that is, a signed value corresponding to the difference between the prior expected reward and the actually obtained reward^36^,^37^. Several studies have found that the FRN was more negative for outcomes worse than expected (negative prediction error)^37^,^38^. The subjective reports of the participants suggest that unfair offers from friends are more surprising than fair offers and fair offers from strangers are surprising than fair offers from friends. Thus, the differences in expectations of the types of offers from friends and strangers may explain the FRN effect. However, the FRN amplitude changes did not always correspond with the changes in subjective report of surprise. For example, we found no significant effect involving anonymity (certainty about identity vs. uncertainty about identity) on FRN amplitude but a significant anonymity by fairness interaction on self-reported surprise. It is worth noting that we only measured general surprise. It is unclear whether participants were positively or negatively surprised. Thus, it is hard to draw any definitive conclusion about the underlying mechanisms medicating the effects of surprise. More research is needed to better understand the effects of surprise and fairness on FRN amplitude.

We found that private allocation in the uncertainty about identity condition elicited a more positive response than public allocation in the certainty about identity condition for the fair offers. Previous studies on outcome evaluation have indicated that the P300 is related to processes of attentional allocation and/or high-level motivational/affective evaluation^39^. We believe that the more positive P300 response to private-allocation than to public-allocation offers reflects differential distribution of attentional resources to the two types of offers, which had different affective/emotional significance. We also would like to suggest that there are top-down processes associated with the P300. Compared with public-allocation, more attentional resources should be devoted to the elaborative processing of speculating the proposer’s identification when the participants are allocated under the anonymous condition. The P300 is also found to be sensitive to the probability of an outcome^29^,^30^,^31^. It is possible that the P300 also reflects the level of uncertainty associated with the identity of the proposer.

According to the theory of “institutional characteristics” by Bohnet and Frey^8^ , with anonymity one has only purely intrinsic motivation to behave fairly; therefore, participants would feel more distressed and less satisfied with unfair asset distribution when they are allocated under private conditions. The stronger P300 responses to private-allocation offers than to public-allocation offers may suggest that the participants in this study attached more irrational affective significance to the private divisions; however, this kind of negative emotion could be moderated by social distance and personal gain, consistent with the social fairness norm cultured in the individual.

### Conclusion

In the present study, we found that participants were more likely to reject not only unfair offers from strangers compared to friends but also unfair offers under the private-allocation compared to the public-allocation condition. Our neurophysiological data demonstrate that social distance may influence fairness consideration and the FRN, a component associated with the processing of conflict between cognitive and emotional motivation; social distance also appears to differentiate offers allocated by friends and strangers. The FRN was more negative-going for unfair offers than fair offers allocated by strangers; however, it showed an opposite trend for unfair offers provided by friends. On the other hand, the P300 was more positive when the offers were allocated in the uncertainty about identity condition than in the certainty about identity condition, irrespective of the degree of fairness. These results suggest that unfairness evaluation may be composed of two processes with differential neural bases: an early fast conflict detection stage and a lateral slower stage that integrates more complex social contextual factors such as anonymity.

## Methods

### Participants

Among the twenty-three electroencephalo-graph (EEG) participants, two stated in the post-experiment questionnaire that they completely disbelieved the setup of the experiment and another two participants with excessive artifacts in EEG recording were excluded from data analysis, leaving nineteen participants (8 males, mean ± SE, 21.57 ± 0.53 years) for the following analysis. All participants were right-handed and had normal or corrected-to-normal vision. They had no history of neurological or psychiatric disorders. The study was approved by the Ethics Committee of the School of Psychology at South China Normal University and was carried out in accordance with the approved guidelines. All participants gave written, informed consent and were informed of their right to discontinue participation at any time. All participants were paid 30 Yuan (about U.S. $4.50) as basic payment. Participants were informed that additional monetary rewards would be paid according to their performance in the task, although in the end all the participants were paid an extra 20 yuan (about U.S. $3) on top of the basic payment.

### Experimental paradigm

Before the EEG experiment, participants were told that they would play a group game with two of their friends and two strangers and that the purpose of this task was to study how people make decisions in a group. When a trio of same-sex friends came to the laboratory, each of them, as well as each of two of same-sex strangers, was asked to stand against the wall and a picture of each person was taken using a digital camera. The purpose of using photos was to make the experimental setup more realistic, which induced the participants to believe they were playing with four real persons. Then the five persons were told that they would sit in separate rooms to finish a task together through the computer network. After the other four participants were led to other rooms, the EEG participant was asked to complete the Chinese version of two questionnaires, the trust scale^40^ and the inclusion of other in the self (IOS) scale^41^, both in relation to their two friends. The trust scale measures to what extent a particular partner is trustworthy, with the score ranging between 18 (completely untrustworthy) and 126 (completely trustworthy). The IOS measure, with two circles overlapping to different degrees to represent a 7-point Likert scale (ranging from 1 to 7; higher scores indicating more inclusion), assessed the perceived closeness between a particular partner and the agent. We also asked participants to complete the trust scale and IOS scale in relation to a stranger after the experiment. The trust scale and the IOS scale were used as a manipulation check in the current study. These two scales were also used to check the social distance manipulation in a Chinese sample in a previous study^32^.

**Figure 1:**
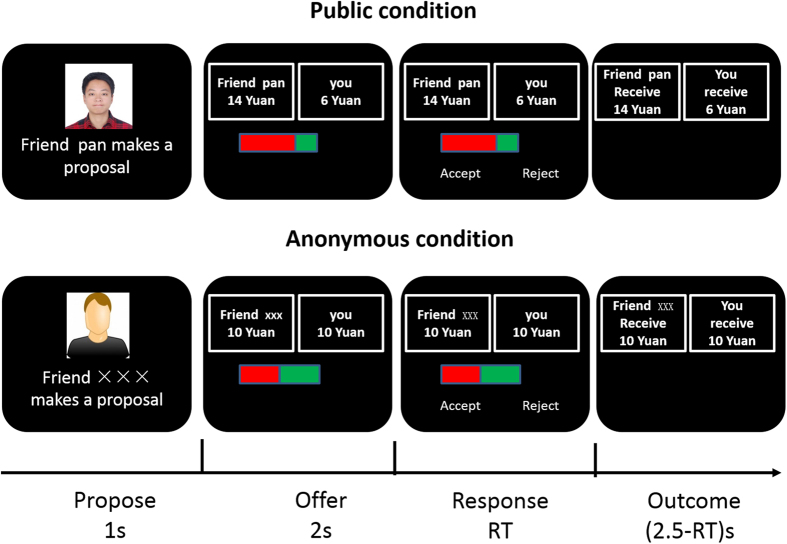
Experimental task design. Participants played the Ultimatum game with friends and strangers. At the beginning of each trial, the name and the picture of the proposer appeared first in the certainty about identity condition. In the uncertainty about identity condition, participants were only informed whether the proposer is one of their friends or one of the strangers. A fair or unfair monetary offer was then presented. After accepting or rejecting, the corresponding outcome was shown.

**Figure 2:**
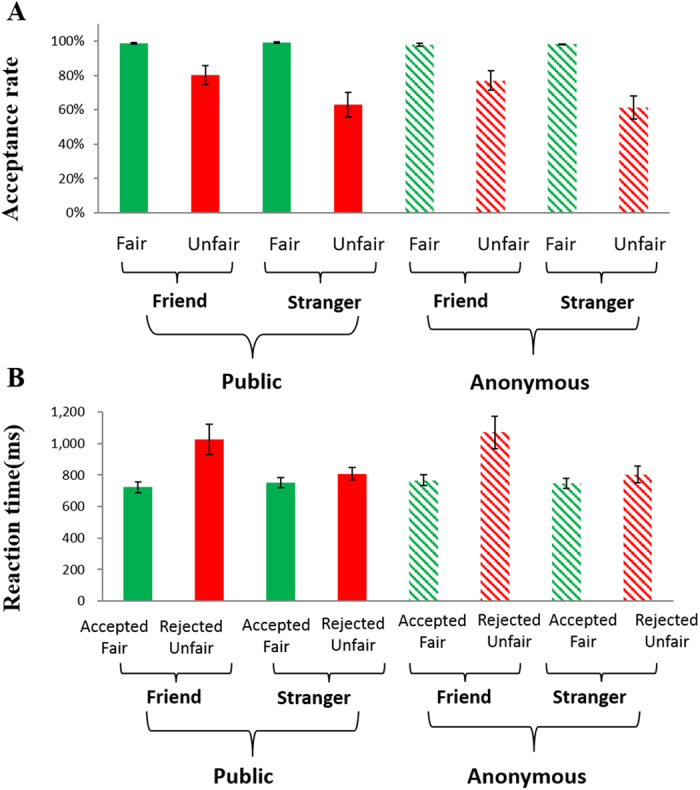
Rate of acceptance and reaction times in experimental conditions. Rate of acceptance (mean ± SE) for the eight experimental conditions (unfair offers in red and fair offers in green) are shown in (**A**). Reaction times (mean ± SE) for the eight experimental conditions are shown in (**B**).

**Figure 3:**
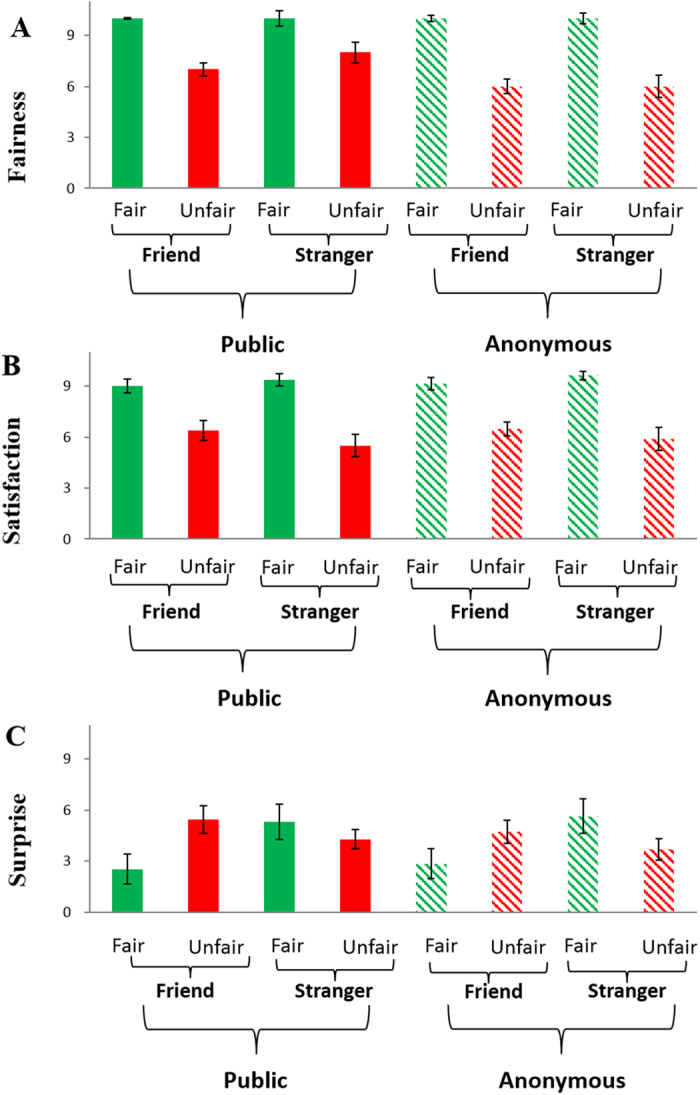
Post-experiment subjective ratings of feeling. The self-reported fairness scores (mean ± SE) for the eight experimental conditions are shown in (**A**). The self-reported satisfaction scores (mean ± SE) for the eight experimental conditions are shown in (**B**). The self-reported surprise scores (mean ± SE) for the eight experimental conditions are shown in (**C**).

**Figure 4:**
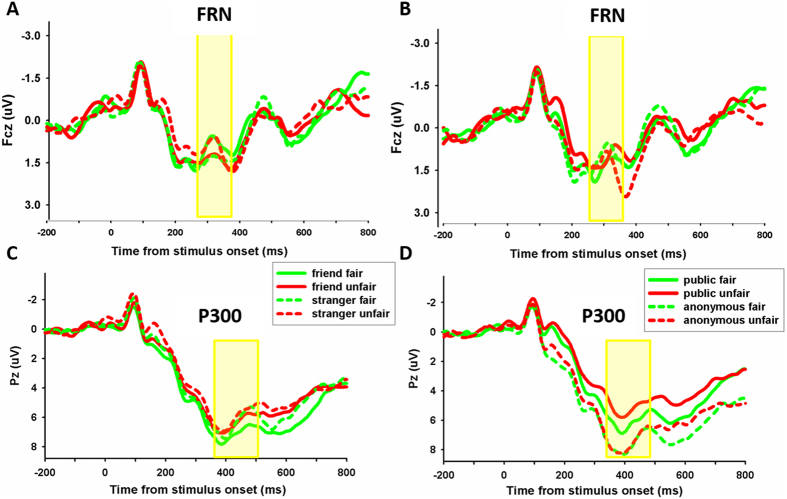
The ERP grand-average waveforms. Grand-average waveforms from channel FCZ using 1–20 HZ band-pass filtered for four experimental conditions (friend-fair, friend-unfair, stranger-fair, stranger-unfair) are shown (**A**), the other four experimental conditions (public-fair, public-unfair, private-fair, private-unfair) are shown (**B**). Grand-average waveforms from channel PZ using 20 Hz low-pass filtered for similar four experimental conditions are shown (**C**,**D**). The shaded 250–350 and 350–500 ms time windows were used to measure the FRN and P300 magnitude, respectively.

**Figure 5:**
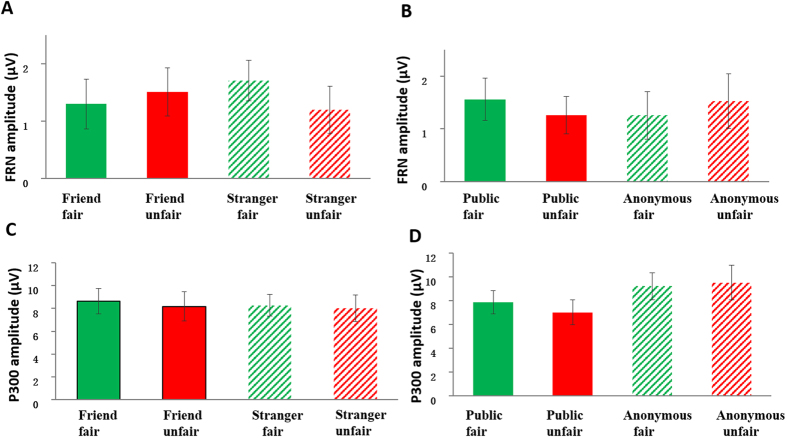
The amplitude of FRN and P300. The FRN amplitudes (mean ± SE, in μV) for the four experimental conditions are shown in (**A**,**B**). The P300 amplitude (mean ± SE, in μV) for the four experimental conditions are shown in (**C**,**D**).

**Figure 6:**
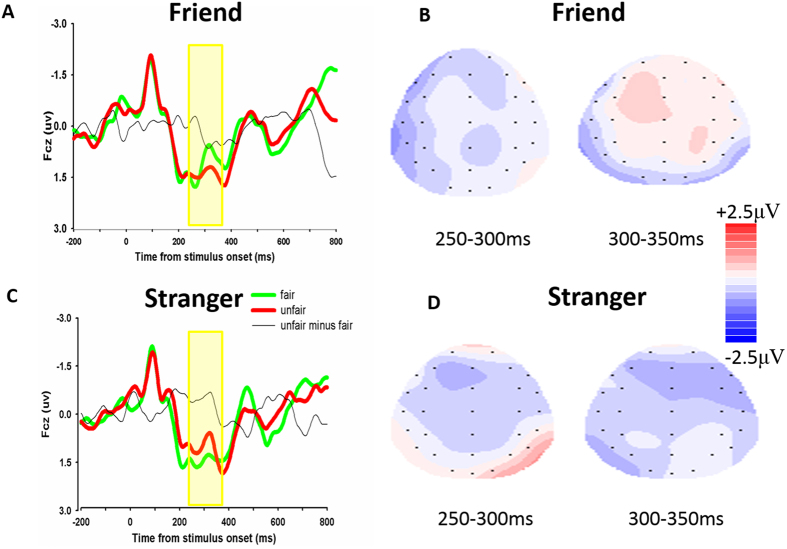
The difference waveforms and topographical maps. Difference waveforms (unfair-fair) and maps of the friend condition are shown in (**A**,**B**). Difference waveforms (unfair-fair) and maps of the stranger condition are shown in (**C**,**D**).

The EEG participants were told that they would play as a recipient in UG and the others would be proposers; in each round of the game, one of their friends or the strangers would receive a 20 Yuan endowment and decide how to divide the amount between the proposer and the participant. The recipient could either accept or reject the proposer’s offer. If accepted, the pie would be divided as proposed; if rejected, both the proposer and recipient would end up empty handed. At the beginning of each round, the picture and the name of the surrogate proposer appeared first (either friend or stranger) for 1s, but the name of the proposer was displayed in one of two ways, either with his/her real name or an anonymous symbol “×××”, which corresponded to a clear photo or an ambiguous one (Fig. 1). This was followed by an offer and a divided color bar that indicated the amount of the offer for 2s. The length of the green portion indicated the amount offered by the proposer, while the red portion indicated the amount retained by the proposer. Participants were required not to respond during this 2s stage. This design allows us to minimize the influence of the button press on brain responses. Participants were required to make a forced choice after the text “Accept” and “Reject” appeared in the screen by pressing the corresponding key within 2.5 seconds. Immediately after their button press response, the corresponding outcome would be shown. The next trial began 0.5 second after the offset of the feedback. Unknown to our participants, the proposals by the surrogate proposer had been arranged ahead of the experiment using the following criteria: 144 trials for fair offers (10:10); 24 trials for each of 6 unfair offers (2:18, 3:17, 4:16, 5:15, 6:14, 7:13), with proposals delivered in a counterbalanced order, for a total of 288 trials. There were 36 trials in each experimental condition. All experimental conditions were randomly presented throughout the sequence. The 288 trials were pseudo-randomized with the restriction that no more than 3 consecutive trials were from the same proposer’s category and no more than 3 consecutive trials were on the same fairness level.

After the EEG session, participants were required to indicate their feeling (satisfaction, fairness or surprise) about eight types of outcomes (fair/unfair proposal from a friend/stranger presented in the identity uncertainty/certainty condition) they experienced in the experiment on a 10-point Likert scale (1 = not at all, 10 = very intensely). Participants were required to report their general surprise regardless whether they are positively or negatively surprised. They were subsequently required to indicate, on a 7-point Likert scale, to what extent they believed the offers were from friends or strangers, with 1 indicating “do not believe at all” and 7 indicating “truly believe”. The participant was debriefed, paid and thanked in the end.

### ERP Recording and Analysis

The participant was seated comfortably about 1.5 m away from the computer screen in a dimly lit and electromagnetically shielded room. The experiment was administered on a Lenovo computer in CRT display, with 1024*768 resolution using E-prime (Psychology Software Tools, Inc. Pittsburgh, PA, www.pstnet.com/eprime) software to control the presentation and timing of stimuli. The EEG was recorded from 64 scalp sites using tin electrodes mounted in an elastic cap (Neuroscan 4.5) according to the International 10–20 system. The vertical-oculogram (VEOG) was recorded from left supra-orbital and infra-orbital electrodes. The horizontal electro-oculogram (HEOG) was recorded from electrodes placed 1.5 cm lateral to the left and right external mastoid. All electrode recordings were referenced to an electrode placed on the left mastoid, and the impedance was maintained below 5 ΚΩ. The electroencephalograph (EEG) and electro-oculogram (EOG) were amplified using a 0.05–70 Hz band-pass and were continuously sampled at 1000 Hz/channel for off-line analysis. The EEG data were re-referenced off-line to linked mastoid electrodes by subtracting from each sample of data recorded at each channel one-half the activity recorded at the right mastoid. Ocular artifacts were corrected with an eye-movement correction algorithm. Epochs of 800 ms (with 200 ms pre-stimulus baseline) EEG for each electrode were time-locked to the onset of offers from the proposers.

The data were then baseline corrected by subtracting from the average activity of that channel during the baseline period. The FRN data were filtered using a 1–20 Hz band-pass (24 dB octave roll off) to remove low-frequency waves from the EEG. The long-duration component P300 is mainly in the frequency range of <3 Hz. Thus, using a 1–20 Hz band-pass can potentially minimize the P300 effect while preserving the FRN effect^42^. The P300 data were filtered using a 20 HZ low pass, consistent with previous research^43^. All trials in which EEG voltages exceeded a threshold of ±70 μν during the recording epoch were excluded from analysis. According to visual inspection of ERP waveforms, the FRN was measured as the mean amplitude in the time window of 250–350 ms post onset of the different offers. The P300 was measured as the peak value in the 350–500 ms time window on each electrode. We focused on the FRN response on the anterior frontal central electrode FCz and the P300 responses on the posterior midline electrode Pz, because the FRN effects and the P300 effects were the largest on these electrodes, respectively.

## Additional Information

**How to cite this article**: Yu, R. *et al.* Social distance and anonymity modulate fairness consideration: An ERP study. *Sci. Rep.* **5**, 13452; doi: 10.1038/srep13452 (2015).
